# 5-Iodo-2-phenyl-3-phenyl­sulfinyl-1-benzofuran

**DOI:** 10.1107/S1600536809024556

**Published:** 2009-07-11

**Authors:** Hong Dae Choi, Pil Ja Seo, Byeng Wha Son, Uk Lee

**Affiliations:** a Department of Chemistry, Dongeui University, San 24 Kaya-dong Busanjin-gu, Busan 614-714, Republic of Korea; b Department of Chemistry, Pukyong National University, 599-1 Daeyeon 3-dong, Nam-gu, Busan 608-737, Republic of Korea

## Abstract

In the title compound, C_20_H_13_IO_2_S, the O atom and the phenyl group of the phenyl­sulfinyl substituent lie on opposite sides of the plane of the benzofuran fragment; the phenyl ring is almost perpendicular to this plane [83.84 (5)°]. The phenyl ring in the 2-position is rotated out of the benzofuran plane, making a dihedral angle of 40.47 (5)°. The crystal structure is stabilized by non-classical inter­molecular C—H⋯O inter­actions, and by an I⋯O halogen bond of 3.124 (1) Å [C—I⋯O = 165.84 (5)°].

## Related literature

For the crystal structures of similar 5-iodo-1-benzofuran derivatives, see: Choi *et al.* (2007*a* ▶,*b* ▶). For a review of halogen inter­actions, see: Politzer *et al.* (2007 ▶). The Cambridge Structural Database (version 5.28; Allen *et al.*, 2002 ▶) has 39 compounds with C–I⋯O=S contact distances less than or equal to 3.3 Å.
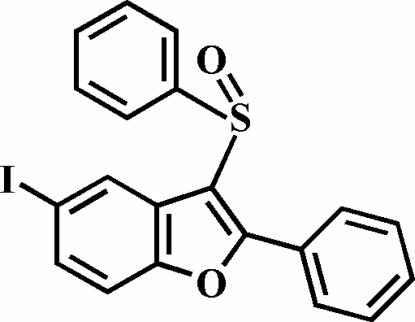


## Experimental

### 

#### Crystal data

C_20_H_13_IO_2_S
*M* *_r_* = 444.26
Triclinic, 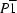

*a* = 9.3544 (4) Å
*b* = 9.7565 (5) Å
*c* = 10.2808 (5) Å
α = 113.381 (1)°
β = 92.640 (1)°
γ = 98.047 (1)°
*V* = 847.42 (7) Å^3^
*Z* = 2
Mo *K*α radiation
μ = 2.02 mm^−1^
*T* = 273 K
0.40 × 0.40 × 0.20 mm

#### Data collection

Bruker SMART CCD diffractometer
Absorption correction: multi-scan (*SADABS*; Sheldrick, 1999 ▶) *T*_min_ = 0.467, *T*_max_ = 0.665
7217 measured reflections
3616 independent reflections
3503 reflections with *I* > 2σ(*I*)
*R*_int_ = 0.014

#### Refinement

*R*[*F* ^2^ > 2σ(*F* ^2^)] = 0.017
*wR*(*F* ^2^) = 0.044
*S* = 1.07
3616 reflections
217 parameters
H-atom parameters constrained
Δρ_max_ = 0.39 e Å^−3^
Δρ_min_ = −0.66 e Å^−3^

### 

Data collection: *SMART* (Bruker, 2001 ▶); cell refinement: *SAINT* (Bruker, 2001 ▶); data reduction: *SAINT*; program(s) used to solve structure: *SHELXS97* (Sheldrick, 2008 ▶); program(s) used to refine structure: *SHELXL97* (Sheldrick, 2008 ▶); molecular graphics: *ORTEP-3* (Farrugia, 1997 ▶) and *DIAMOND* (Brandenburg, 1998 ▶); software used to prepare material for publication: *SHELXL97*.

## Supplementary Material

Crystal structure: contains datablocks global, I. DOI: 10.1107/S1600536809024556/ng2605sup1.cif

Structure factors: contains datablocks I. DOI: 10.1107/S1600536809024556/ng2605Isup2.hkl

Additional supplementary materials:  crystallographic information; 3D view; checkCIF report

## Figures and Tables

**Table 1:** Hydrogen-bond geometry (Å, °)

*D*—H⋯*A*	*D*—H	H⋯*A*	*D*⋯*A*	*D*—H⋯*A*
C10—H10⋯O2^i^	0.93	2.53	3.438 (2)	165
C19—H19⋯O1^ii^	0.93	2.59	3.483 (2)	162
C20—H20⋯O2^iii^	0.93	2.53	3.412 (2)	159

Symmetry codes: (i) 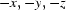; (ii) 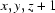; (iii) 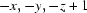.

**Fig. 1.**
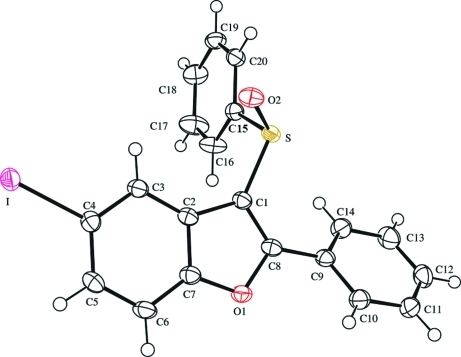
The molecular structure of the title compound with the atom numbering scheme. Displacement ellipsoids are drawn at the 50% probability level. H atoms are presented as a small spheres of arbitrary radius.

**Fig. 2.**
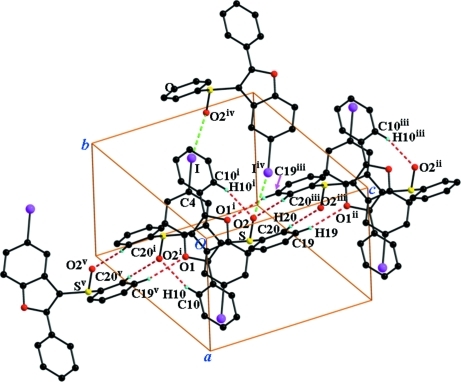
The C–H···O hydrogen bond and I···O halogen bond (dotted lines) in the title compound. [Symmetry code: (i) -x, -y, -z; (ii) x, y, z + 1; (iii) -x, -y, -z + 1; (iv) -x, -y + 1, -z + 1; (v) x, y, z - 1.]

## supplementary crystallographic information

### Comment

This work is related to our communications on the synthesis and structures of 5-iodo-1-benzofuran analogues, viz. 5-iodo-3-methylsulfinyl-2-phenyl-1-benzofuran (Choi *et al.*,  2007*a*) and 5-iodo-2-methyl-3-phenylsulfinyl-1-benzofuran (Choi *et al.*,  2007*b*). We present the crystal structure of the title compound (I), 5-iodo-2-phenyl-3-phenylsulfinyl-1-benzofuran (Fig. 1).

The benzofuran unit is essentially planar, with a mean deviation of 0.005 (1) Å from the least-squares plane defined by the nine constituent atoms. The dihedral angle in (I) formed by the planes of the benzofuran and 2-phenyl rings is 40.47 (5)°, and the phenyl ring (C15-C20) with 83.84 (5)° lies toward the benzofuran plane. The crystal packing (Fig. 2) is stabilized by non-classical intermolecular C–H···O interactions; the first between an H atom of the 2-phenyl ring and the oxygen of the S═O unit, with a C10–H10···O2^i^, the second between a phenyl H atom of the phenylsulfinyl substituent and the furan O atom, with a C19–H19···O1^ii^, the third between a phenyl H atom of the phenylsulfinyl substituent and the oxygen of the S═O unit, with a C20–H20···O2^iii^, respectively (Table 1 and Fig. 2; symmetry code as in Fig. 2). The molecular packing (Fig. 2) is further stabilized by an I···O halogen bond (Politzer *et al.*,  2007) between the iodine atom and the oxygen of neighbouring S═O unit, with an I···O^iv^ (Symmetry code as in Fig. 2). The observed I···O separation of 3.124 (1) Å and the nearly linear C–I···O angle of 165.84 (5)° are typical for such halogen bonds. A search of Cambridge Structural Database (version 5.28; Allen, 2002) revealed 39 compounds with C–I···O═S contact distances equal to or less than 3.3 Å.

### Experimental

The 77% 3-chloroperoxybenzoic acid (123 mg, 0.55 mmol) was added in small portions to a stirred solution of 5-iodo-2-phenyl-3-phenylsulfanyl-1-benzofuran (214 mg, 0.5 mmol) in dichloromethane (30 mL) at 273 K. After being stirred at room temperature for 3h, the mixture was washed with saturated sodium bicarbonate solution and the organic layer was separated, dried over magnesium sulfate, filtered and concentrated in vacuum. The residue was purified by column chromatography (hexane-ethyl acetate, 2:1 v/v) to afford the title compound as a colorless solid [yield 76%, m.p. 423-424 K; *R*_f_ = 0.74 (hexane-ethyl acetate, 2:1 v/v)]. Single crystals suitable for X-ray diffraction were prepared by slow evaporation of a solution of the title compound in acetone at room temperature.

### Refinement

All H atoms were positioned geometrically and refined using a riding model, with C–H = 0.93 Å for aromatic H atoms and with Uiso(H) = 1.2*U*eq(C) for aromatic H atoms.

### Crystal data

**Table:**

C_20_H_13_IO_2_S	*Z* = 2
*M**_r_* = 444.26	*F*(000) = 436
Triclinic, *P*1	*D*_x_ = 1.741 Mg m^−^^3^
Hall symbol: -P 1	Mo *K*α radiation, λ = 0.71073 Å
*a* = 9.3544 (4) Å	Cell parameters from 6672 reflections
*b* = 9.7565 (5) Å	θ = 2.2–27.5°
*c* = 10.2808 (5) Å	µ = 2.02 mm^−^^1^
α = 113.381 (1)°	*T* = 273 K
β = 92.640 (1)°	Block, colorless
γ = 98.047 (1)°	0.40 × 0.40 × 0.20 mm
*V* = 847.42 (7) Å^3^	

### Data collection

**Table:**

Bruker SMART CCD diffractometer	3616 independent reflections
Radiation source: fine-focus sealed tube	3503 reflections with *I* > 2σ(*I*)
graphite	*R*_int_ = 0.014
Detector resolution: 10.0 pixels mm^-1^	θ_max_ = 27.0°, θ_min_ = 2.2°
φ and ω scans	*h* = −11→11
Absorption correction: multi-scan (*SADABS*; Sheldrick, 1999)	*k* = −12→12
*T*_min_ = 0.467, *T*_max_ = 0.665	*l* = −13→12
7217 measured reflections	

### Refinement

**Table:**

Refinement on *F*^2^	Primary atom site location: structure-invariant direct methods
Least-squares matrix: full	Secondary atom site location: difference Fourier map
*R*[*F*^2^ > 2σ(*F*^2^)] = 0.017	Hydrogen site location: difference Fourier map
*wR*(*F*^2^) = 0.044	H-atom parameters constrained
*S* = 1.07	*w* = 1/[σ^2^(*F*_o_^2^) + (0.0224*P*)^2^ + 0.4472*P*] where *P* = (*F*_o_^2^ + 2*F*_c_^2^)/3
3616 reflections	(Δ/σ)_max_ < 0.001
217 parameters	Δρ_max_ = 0.39 e Å^−^^3^
0 restraints	Δρ_min_ = −0.66 e Å^−^^3^

### Special details

**Table:**

Geometry. All esds (except the esd in the dihedral angle between two l.s. planes) are estimated using the full covariance matrix. The cell esds are taken into account individually in the estimation of esds in distances, angles and torsion angles; correlations between esds in cell parameters are only used when they are defined by crystal symmetry. An approximate (isotropic) treatment of cell esds is used for estimating esds involving l.s. planes.
Refinement. Refinement of F^2^ against ALL reflections. The weighted R-factor wR and goodness of fit S are based on F^2^, conventional R-factors R are based on F, with F set to zero for negative F^2^. The threshold expression of F^2^ > 2sigma(F^2^) is used only for calculating R-factors(gt) etc. and is not relevant to the choice of reflections for refinement. R-factors based on F^2^ are statistically about twice as large as those based on F, and R- factors based on ALL data will be even larger.

### Fractional atomic coordinates and isotropic or equivalent isotropic displacement parameters (Å^2^)

**Table:**

	*x*	*y*	*z*	*U*_iso_*/*U*_eq_	
I	0.041308 (11)	0.682844 (11)	0.391884 (12)	0.02610 (5)	
S	0.15151 (4)	0.00846 (5)	0.27065 (4)	0.02113 (8)	
O1	0.23863 (13)	0.11727 (14)	−0.05193 (12)	0.0244 (2)	
O2	0.01344 (13)	0.05325 (15)	0.32919 (13)	0.0278 (3)	
C1	0.19488 (18)	0.09925 (19)	0.15550 (17)	0.0213 (3)	
C2	0.16806 (17)	0.24593 (19)	0.16717 (17)	0.0212 (3)	
C3	0.12402 (18)	0.37088 (19)	0.27157 (18)	0.0229 (3)	
H3	0.1038	0.3714	0.3595	0.027*	
C4	0.11164 (18)	0.49421 (19)	0.23915 (18)	0.0231 (3)	
C5	0.14158 (19)	0.4962 (2)	0.10730 (19)	0.0263 (3)	
H5	0.1329	0.5815	0.0901	0.032*	
C6	0.1841 (2)	0.3719 (2)	0.00232 (19)	0.0274 (4)	
H6	0.2037	0.3707	−0.0860	0.033*	
C7	0.19590 (18)	0.24964 (19)	0.03641 (18)	0.0231 (3)	
C8	0.23701 (18)	0.02755 (19)	0.02314 (17)	0.0222 (3)	
C9	0.28197 (18)	−0.11933 (19)	−0.05014 (18)	0.0223 (3)	
C10	0.23677 (19)	−0.2044 (2)	−0.19558 (19)	0.0265 (3)	
H10	0.1800	−0.1671	−0.2463	0.032*	
C11	0.2777 (2)	−0.3454 (2)	−0.2632 (2)	0.0315 (4)	
H11	0.2461	−0.4036	−0.3592	0.038*	
C12	0.3653 (2)	−0.4000 (2)	−0.1890 (2)	0.0347 (4)	
H12	0.3929	−0.4939	−0.2355	0.042*	
C13	0.4117 (2)	−0.3148 (2)	−0.0455 (2)	0.0344 (4)	
H13	0.4706	−0.3515	0.0041	0.041*	
C14	0.3700 (2)	−0.1747 (2)	0.0242 (2)	0.0286 (4)	
H14	0.4009	−0.1178	0.1206	0.034*	
C15	0.29386 (18)	0.10909 (19)	0.41498 (17)	0.0216 (3)	
C16	0.4258 (2)	0.1827 (2)	0.3995 (2)	0.0333 (4)	
H16	0.4411	0.1914	0.3142	0.040*	
C17	0.5347 (2)	0.2429 (3)	0.5131 (2)	0.0412 (5)	
H17	0.6233	0.2931	0.5041	0.049*	
C18	0.5124 (2)	0.2288 (2)	0.6400 (2)	0.0342 (4)	
H18	0.5864	0.2684	0.7152	0.041*	
C19	0.3803 (2)	0.1560 (2)	0.65465 (19)	0.0287 (4)	
H19	0.3655	0.1473	0.7400	0.034*	
C20	0.26963 (19)	0.09587 (19)	0.54241 (18)	0.0244 (3)	
H20	0.1805	0.0474	0.5523	0.029*	

### Atomic displacement parameters (Å^2^)

**Table:**

	*U*^11^	*U*^22^	*U*^33^	*U*^12^	*U*^13^	*U*^23^
I	0.02785 (7)	0.02109 (7)	0.03128 (7)	0.00645 (4)	0.00755 (5)	0.01148 (5)
S	0.02433 (19)	0.02144 (19)	0.02157 (18)	0.00522 (15)	0.00503 (15)	0.01221 (15)
O1	0.0307 (6)	0.0269 (6)	0.0205 (5)	0.0099 (5)	0.0065 (5)	0.0129 (5)
O2	0.0216 (6)	0.0364 (7)	0.0300 (6)	0.0054 (5)	0.0069 (5)	0.0178 (6)
C1	0.0226 (7)	0.0228 (8)	0.0218 (7)	0.0055 (6)	0.0034 (6)	0.0120 (6)
C2	0.0206 (7)	0.0244 (8)	0.0222 (7)	0.0045 (6)	0.0026 (6)	0.0130 (6)
C3	0.0241 (8)	0.0257 (8)	0.0228 (8)	0.0058 (6)	0.0054 (6)	0.0133 (7)
C4	0.0213 (8)	0.0225 (8)	0.0268 (8)	0.0049 (6)	0.0042 (6)	0.0107 (7)
C5	0.0285 (8)	0.0267 (9)	0.0306 (9)	0.0068 (7)	0.0027 (7)	0.0182 (7)
C6	0.0324 (9)	0.0342 (9)	0.0242 (8)	0.0100 (7)	0.0064 (7)	0.0190 (7)
C7	0.0235 (8)	0.0271 (8)	0.0223 (8)	0.0071 (6)	0.0038 (6)	0.0129 (7)
C8	0.0223 (7)	0.0264 (8)	0.0219 (8)	0.0052 (6)	0.0022 (6)	0.0137 (7)
C9	0.0215 (7)	0.0250 (8)	0.0228 (8)	0.0045 (6)	0.0063 (6)	0.0115 (7)
C10	0.0267 (8)	0.0279 (9)	0.0245 (8)	0.0028 (7)	0.0032 (7)	0.0108 (7)
C11	0.0371 (10)	0.0266 (9)	0.0260 (9)	−0.0001 (7)	0.0091 (7)	0.0070 (7)
C12	0.0409 (11)	0.0231 (9)	0.0413 (11)	0.0096 (8)	0.0169 (9)	0.0118 (8)
C13	0.0364 (10)	0.0333 (10)	0.0414 (11)	0.0150 (8)	0.0092 (8)	0.0201 (9)
C14	0.0316 (9)	0.0296 (9)	0.0266 (8)	0.0098 (7)	0.0038 (7)	0.0121 (7)
C15	0.0225 (8)	0.0233 (8)	0.0227 (8)	0.0081 (6)	0.0039 (6)	0.0117 (6)
C16	0.0260 (9)	0.0531 (12)	0.0292 (9)	0.0036 (8)	0.0057 (7)	0.0262 (9)
C17	0.0238 (9)	0.0654 (15)	0.0401 (11)	−0.0027 (9)	0.0013 (8)	0.0312 (11)
C18	0.0286 (9)	0.0472 (11)	0.0289 (9)	0.0068 (8)	−0.0010 (7)	0.0179 (8)
C19	0.0370 (10)	0.0330 (9)	0.0218 (8)	0.0109 (8)	0.0069 (7)	0.0151 (7)
C20	0.0272 (8)	0.0239 (8)	0.0264 (8)	0.0071 (6)	0.0079 (7)	0.0133 (7)

### Geometric parameters (Å, °)

**Table:**

I—C4	2.104 (2)	C10—C11	1.391 (3)
I—O2^i^	3.124 (1)	C10—H10	0.9300
S—O2	1.497 (1)	C11—C12	1.387 (3)
S—C1	1.768 (2)	C11—H11	0.9300
S—C15	1.799 (2)	C12—C13	1.386 (3)
O1—C8	1.377 (2)	C12—H12	0.9300
O1—C7	1.378 (2)	C13—C14	1.389 (3)
C1—C8	1.367 (2)	C13—H13	0.9300
C1—C2	1.447 (2)	C14—H14	0.9300
C2—C7	1.394 (2)	C15—C16	1.388 (2)
C2—C3	1.397 (2)	C15—C20	1.392 (2)
C3—C4	1.388 (2)	C16—C17	1.387 (3)
C3—H3	0.9300	C16—H16	0.9300
C4—C5	1.404 (2)	C17—C18	1.386 (3)
C5—C6	1.389 (3)	C17—H17	0.9300
C5—H5	0.9300	C18—C19	1.382 (3)
C6—C7	1.387 (2)	C18—H18	0.9300
C6—H6	0.9300	C19—C20	1.389 (3)
C8—C9	1.463 (2)	C19—H19	0.9300
C9—C14	1.397 (2)	C20—H20	0.9300
C9—C10	1.401 (2)		
			
C4—I—O2^i^	165.84 (5)	C11—C10—H10	120.4
O2—S—C1	107.63 (7)	C9—C10—H10	120.4
O2—S—C15	106.00 (8)	C12—C11—C10	120.66 (17)
C1—S—C15	100.57 (8)	C12—C11—H11	119.7
C8—O1—C7	106.38 (12)	C10—C11—H11	119.7
C8—C1—C2	107.02 (14)	C13—C12—C11	120.13 (17)
C8—C1—S	123.64 (13)	C13—C12—H12	119.9
C2—C1—S	128.38 (12)	C11—C12—H12	119.9
C7—C2—C3	119.26 (15)	C12—C13—C14	119.94 (18)
C7—C2—C1	104.91 (14)	C12—C13—H13	120.0
C3—C2—C1	135.83 (15)	C14—C13—H13	120.0
C4—C3—C2	117.47 (15)	C13—C14—C9	120.11 (17)
C4—C3—H3	121.3	C13—C14—H14	119.9
C2—C3—H3	121.3	C9—C14—H14	119.9
C3—C4—C5	122.37 (16)	C16—C15—C20	120.95 (16)
C3—C4—I	118.65 (12)	C16—C15—S	123.40 (13)
C5—C4—I	118.96 (12)	C20—C15—S	115.34 (13)
C6—C5—C4	120.55 (15)	C17—C16—C15	119.05 (17)
C6—C5—H5	119.7	C17—C16—H16	120.5
C4—C5—H5	119.7	C15—C16—H16	120.5
C7—C6—C5	116.34 (16)	C18—C17—C16	120.50 (18)
C7—C6—H6	121.8	C18—C17—H17	119.8
C5—C6—H6	121.8	C16—C17—H17	119.8
O1—C7—C6	125.17 (15)	C19—C18—C17	120.03 (18)
O1—C7—C2	110.82 (14)	C19—C18—H18	120.0
C6—C7—C2	124.00 (16)	C17—C18—H18	120.0
C1—C8—O1	110.87 (14)	C18—C19—C20	120.30 (16)
C1—C8—C9	133.30 (15)	C18—C19—H19	119.8
O1—C8—C9	115.81 (14)	C20—C19—H19	119.8
C14—C9—C10	119.94 (16)	C19—C20—C15	119.15 (16)
C14—C9—C8	120.26 (15)	C19—C20—H20	120.4
C10—C9—C8	119.80 (15)	C15—C20—H20	120.4
C11—C10—C9	119.19 (17)		
			
O2—S—C1—C8	−134.10 (15)	C7—O1—C8—C1	−0.06 (18)
C15—S—C1—C8	115.21 (15)	C7—O1—C8—C9	−178.86 (14)
O2—S—C1—C2	33.18 (17)	C1—C8—C9—C14	−39.3 (3)
C15—S—C1—C2	−77.52 (16)	O1—C8—C9—C14	139.16 (16)
C8—C1—C2—C7	0.54 (18)	C1—C8—C9—C10	140.9 (2)
S—C1—C2—C7	−168.40 (13)	O1—C8—C9—C10	−40.7 (2)
C8—C1—C2—C3	−179.78 (19)	C14—C9—C10—C11	1.5 (3)
S—C1—C2—C3	11.3 (3)	C8—C9—C10—C11	−178.63 (16)
C7—C2—C3—C4	−0.6 (2)	C9—C10—C11—C12	−1.6 (3)
C1—C2—C3—C4	179.73 (18)	C10—C11—C12—C13	0.7 (3)
C2—C3—C4—C5	0.0 (2)	C11—C12—C13—C14	0.2 (3)
C2—C3—C4—I	178.71 (12)	C12—C13—C14—C9	−0.2 (3)
C3—C4—C5—C6	0.6 (3)	C10—C9—C14—C13	−0.7 (3)
I—C4—C5—C6	−178.10 (13)	C8—C9—C14—C13	179.52 (17)
C4—C5—C6—C7	−0.5 (3)	O2—S—C15—C16	−135.76 (16)
C8—O1—C7—C6	179.37 (17)	C1—S—C15—C16	−23.80 (17)
C8—O1—C7—C2	0.42 (18)	O2—S—C15—C20	50.62 (14)
C5—C6—C7—O1	−178.91 (16)	C1—S—C15—C20	162.58 (13)
C5—C6—C7—C2	−0.1 (3)	C20—C15—C16—C17	0.3 (3)
C3—C2—C7—O1	179.66 (14)	S—C15—C16—C17	−172.99 (17)
C1—C2—C7—O1	−0.59 (18)	C15—C16—C17—C18	0.5 (3)
C3—C2—C7—C6	0.7 (3)	C16—C17—C18—C19	−0.8 (4)
C1—C2—C7—C6	−179.56 (16)	C17—C18—C19—C20	0.4 (3)
C2—C1—C8—O1	−0.30 (19)	C18—C19—C20—C15	0.4 (3)
S—C1—C8—O1	169.29 (11)	C16—C15—C20—C19	−0.7 (3)
C2—C1—C8—C9	178.21 (17)	S—C15—C20—C19	173.06 (13)
S—C1—C8—C9	−12.2 (3)		

Symmetry codes: (i) −*x*, −*y*+1, −*z*+1.

### Hydrogen-bond geometry (Å, °)

**Table:**

*D*—H···*A*	*D*—H	H···*A*	*D*···*A*	*D*—H···*A*
C10—H10···O2^ii^	0.93	2.53	3.438 (2)	165
C19—H19···O1^iii^	0.93	2.59	3.483 (2)	162
C20—H20···O2^iv^	0.93	2.53	3.412 (2)	159

Symmetry codes: (ii) −*x*, −*y*, −*z*; (iii) *x*, *y*, *z*+1; (iv) −*x*, −*y*, −*z*+1.
